# Hospital Meetings

**Published:** 1904-03-12

**Authors:** 


### HOSPITAL MEETINGS.

### LONDON HOSPITAL.

A special general Court of Governors was held on March 2nd at this hospital. Owing to the inclemency of the weather, there was some difficulty at first in forming a quorum, but, this having been surmounted, the proceedings began under the presidency of Mr. J. H. Hale. The Secretary read the report of the House Committee for the past quarter, which suggested the nomination of Mr. J. H. Buxton as Vice-President of the hospital, this gentleman having recently resigned the post of treasurer. This motion was put to the meeting and passed unanimously. The report went on to state that great progress had been made in the new buildings; the new laundry was now open, and the new kitchens had been open for a month; it was hoped that the basement containing ophthalmic wards would be ready for use in a fortnight. Mr. James Hora had given the sum of £5,000 towards the maternity wards. A petition had been made to the Borough Council that the roadway adjoining the new isolation block at the rear of the hospital might be paved with wood, which would much lessen the suffering of the patients. With regard to the Bill for the State Registration of Nurses, the committee were opposing it by all the means in their power. The hospital had decided to work with the Cancer Research Fund, and would supply specimens of any material they might require. Under the will of the late Mr. Dresden they hoped to receive the sum of £6,800. The past year had been one of extremely hard work in connection with the quinquennial appeal, which had resulted in the total of £138,000, or, together with the gifts of stock, £140,000. The patients discharged cured during the last quarter numbered 369, relieved 2,400, died 300. The highest number of patients on any one day was 734.

The Chairman, in moving the adoption of the report, congratulated the hospital on the fact that throughout the whole of the rebuilding operations it had never been found necessary to close it for one single day. The rebuilding would not be finished for another 18 months. During the last week they had had a record day for out-patients, the number amounting to 1,501. Operations under anæsthetics had increased from 10,000 in 1902 to 14,000 in 1903. Lupus cases were daily increasing; these took a considerable time for treatment, so that a new patient put on the books at the present time could not be treated for two and a half years.

The general meeting was, after the adoption of the report, made into an extraordinary meeting for the purpose of affixing the hospital seal to various leases.

### THE MIDDLESEX HOSPITAL.

The annual Court of Governors, at which the Earl of Lathom presided, was held at the Middlesex Hospital on the 25th ult.

The Court had before them two separate reports, the one dealing with the work of the hospital and its convalescent home branch at Clacton-on-Sea, and the other that of the Cancer Charity. From the former is gleaned the fact that all the various building improvements which have been in course of construction during the last five years are now complete, and the hospital may be said to be equipped with every known appliance for the treatment of its patients. These various alterations during the last decade have comprised, *inter alia*, the enlargement of some of the female wards, the building and equipment of a separate department (with a special operating-theatre) for the treatment of diseases of women, the erection of a new home for the nursing staff, the removal of the hospital kitchens from the basement to the top floor, the removal of the laundry from the hospital to a site in the country, the installation of a new boiler-house, the erection of a refrigerating and cold-storage plant, and disinfecting apparatus.

The cost of all these alterations has been considerable, so that the hospital now finds itself, after having realised a considerable amount of its capital, still in debt to the bankers to the extent of £20,000. With the object of repaying this debt a festival dinner is to be held in May next. With regard to the work of last year, the statistics show that 4,095 in-patients and 48,744 out-patients, as well as 272 patients who attended for the "light" cure, were treated at the hospital, and 979 patients were sent to the hospital's home at Clacton-on-Sea.

The second report submitted—that of the Cancer Charity—is the first of its kind. After dealing with the financial affairs of the Charity it gives the statistics of the patients treated. It mentions the fact that the Charity is now regarded as a separate institution by both the Hospital Sunday and Saturday Funds, who make awards accordingly. Dealing with the treatment of the patients, mention is made that 100 cases of carcinoma were treated with 3,040 applications of $x$-rays with distinctly encouraging results, that in the laboratories much time had been devoted to the testing of alleged "cancer cures," and no so-called cure submitted had been neglected, but all had as yet proved fruitless.

The director of the laboratories, Dr. Lazarus-Barlow, has been appointed a member of the German Cancer

Investigation Committee, so that the two bodies are able to work together, and a much happier result may be anticipated from the combination.

### ROYAL LONDON OPHTHALMIC HOSPITAL.

The annual Court of Governors was held on February 25th. Lord Avebury had hoped to take the chair, but was unfortunately confined to the house by a severe cold, his place being filled by Mr. H. P. Sturgis, chairman of the committee of management. There was a good attendance of governors, chiefly ladies.

The adoption of the report for 1903 was moved by the chairman, who said that the most important fact of the past year had been the opening of 30 additional beds in the hospital. This they had been able to do through the generosity and assistance of King Edward's Hospital Fund, from whom they had received a large grant (£4,500), conditional on their opening the extra beds. They had read with great pleasure the statement made by the committee of the Fund, that in their opinion the hospital should receive more support from the public. This was extremely gratifying to them, both as a compliment and as showing that the committee of the Fund (who always made a careful examination into the management of the hospitals) were perfectly content with their management. There had been a great increase in the work of the hospital during the past year, the daily average of beds occupied being 98, as against 74 in 1902, and this number would have been larger but for outbreaks of measles. It had also been a record year in the out-patients' department, where the new out-patients numbered 37,987. The increase in the number of beds had thrown more work upon the staff and everyone connected with the hospital, but owing to their good system of organisation no dislocation of the work had taken place, but all had gone smoothly. They were glad to note that the increase in the annual subscriptions continued, but donations and legacies had fallen off. There had been an increase in expenditure, but this was almost entirely due to the opening of the additional beds, and it was satisfactory to note that the estimated cost of the beds had not been exceeded. It was customary when examining the working of a hospital to take into consideration the cost of beds; but this was an unfair criterion in the case of special hospitals, who did the bulk of their work with out-patients and had but a small number of beds. The expenditure had exceeded income by £1,438, making a total deficiency of £6,901. Of this sum £4,500 represented repayment of the balance of the loan they had borrowed from the Charity Commissioners. They were making a great appeal this year, being their centenary year, for assistance towards the repayment of this loan, also for a sum of £6,000 in further annual subscriptions or donations to meet the annual expenses, and finally for £50,000 for the rent fund investment, towards meeting the heavy ground rent of £1,210 a year. They had £1,170 towards this fund already. They were very grateful to the Royal London Ophthalmic Guild for the very valuable assistance it had rendered in the past year in providing all the extra linen, blankets, etc., for the 30 new beds and for the special donation of £100 for the maintenance of one bed and one cot. The committee much regretted the loss of the services of their most excellent matron, Miss Richards, who had left them to become one of the Queen's Nurses, and they were glad that they had been able to supply her place by one of her own sisters, Miss Cook, who they trusted would maintain the high traditions of her predecessor.

Mr. A. Gordon Pollock, in seconding the resolution, alluded to the grievous burden the hospital had to bear in the way of rates, which amounted last year to £936, being much heavier than that of most hospitals in London, St. Thomas's and Guy's alone paying larger amounts.

Among the votes of thanks that followed was one to the nursing staff, moved by Mr. John Tweedy, who dwelt at some length upon the immense importance of skilled nursing, especially in an eye hospital, and said how glad he was to feel that as the science of medicine and surgery advanced, so also did the nursing.

A vote of thanks to the chairman terminated the proceedings.

### CANCER HOSPITAL, FULHAM ROAD.

In the absence of Lord Ludlow, the chair was taken at the annual meeting of governors on February 25th by Lieut.-Col. Parr. The adoption of the report was moved by the Chairman, who referred to the loss sustained by the resignation of Miss Rogers, the Matron, who had done such excellent work for many years, and congratulated the hospital on having obtained the services of so capable a successor. The committee were glad to be able to say that they had reduced their expenses during the past year; a committee had been formed and had gone into the question of expenditure very carefully, with the result that they had been able to reduce the expenses under several headings. On the other hand, however, they had been obliged to undertake further expenses in connection with the electrical department and light treatment. By the generosity of an anonymous friend they were making a very thorough trial of the treatment by radium, and systematic records were being kept of the results. He claimed for himself the credit of being the first to introduce the question as to the desirability of erecting a new home for the nurses, which should be separated from the hospital. This home it was now decided to build in rear of the hospital grounds at a cost of about £5,000. To meet this outlay they would be compelled to dip into capital, but they were convinced of the necessity of the undertaking and of the benefit it would be to the nurses to have their home apart from the hospital.

The report was adopted, and various votes of thanks were then passed.

In reply to the vote of thanks to the medical staff, Dr. Herbert L. Snow, senior surgeon, said that any dispassionate observer who surveyed the career of that hospital would be specially struck by its conspicuous honesty. It had always done its work by legitimate means, and had resolutely refused to ally itself with any of the impostures and bogus remedies or discoveries so rife in that particular department of medical art. Numerous surgical operations for the cure or relief of cancer had either originated or had been mainly perfected there. They had studied "cancer" as, to the best of his knowledge, it had been studied nowhere else in the world. They had ascertained numerous facts and principles; had published these, often at huge pecuniary cost to their individual selves, and had endeavoured to procure for them due recognition among their medical brethren. In short, they had laboured by every means in their power to substitute for the mass of chaotic confusion and traditional fallacy which had hitherto prevailed—and of which the very word "cancer" itself presented an apt example—a genuine and precise cancer-science, as a sure foundation for all future research. In this attempt they had failed—utterly and miserably failed. And now on all hands they found schemes of cancer research, one under highly august patronage, and their own, in the able hands of Mr. Plimmer, not the least promising. These also must fail—must of necessity fail—unless or until some step was taken to clear away all the traditional lumber of past centuries, and to formulate distinctly and precisely what was known with certainty, what was known only partially and imperfectly, and what yet remained to be ascertained. At present all was groping in the dark —or little more. In his humble opinion, a World's Congress on cancer was the one thing needful. With such august and wealthy patronage such a suggestion was surely not altogether utopian, but might be regarded as fairly within the limit of practical politics.

Mr. F. Bowreman Jessett took exception to the use of the word "experiment" by Dr. Snow, and denied that experiments were made in that hospital. When patients came to them in the early stages of the disease they were cured; he knew of cases of patients who had been treated 12 and 14 years ago who were now quite well. On the question of expenditure he wished to say that the expenses were high because a certain number of their patients stopped in the hospital for the remainder of their lives and required expensive medicines and an enormous amount of nursing. He said this because the Hospital Sunday Fund considered their expenses too high, but he thought these facts should be taken into consideration.

The subscriptions and donations of the last year showed an increase; of these £200 came from King Edward's Hospital Fund, £325 from the Sunday Fund, and £193 from the Saturday Fund. The committee have decided upon the erection of a passenger lift, which it is hoped will prove of inestimable benefit to the patients. The number of new in-patients was 711, and of out-patients 1,714. The average number of beds occupied was 84; 501 operations were performed, and the average rate of mortality was only 3·14 per cent.

### THE EVELINA HOSPITAL: A WARD RE-NAMED.

One of the hospitals selected by the Trustees of the Zunz Bequest, which, it will be remembered, was to be awarded to Metropolitan hospitals, at their discretion, is the Evelina Hospital for Sick Children. On the strength of their donation, the authorities of the hospital have recently been carrying out certain much needed improvements, and on Wednesday afternoon, 24th ult, in accordance with a condition of the legacy, a ward was renamed the Annie Zunz Ward. The ceremony was performed by Mrs. Leopold de Rothschild, in the presence of a large number of visitors. The ward in question—a surgical ward—was prettily decorated with red tulips and white lilac, while tall palms and banks of azaleas were placed at the entrance. Mrs. de Rothschild, upon arrival, was presented with a bouquet of lilies of the valley, by a little patient who also recited some verses of welcome.

The Chairman of the Hospital, Mr. Charles Wightman, said the trustees of the Zunz Bequest had generously awarded the sum of £5,000 to the hospital, and the Committee was most grateful for their kindness. He regretted that neither of the trustees were able to be present on the occasion, and also that the President of the hospital, Lord Ancaster, was unavoidably absent. He wished very warmly to thank Mrs. de Rothschild, not only for her presence there that day, but for her constant interest in the welfare of the institution. It was chiefly at her instigation that some of the most important improvements had been carried out, and among these he must mention the new quarters for the nurses. He had always thought that hospital nurses ought to be especially well cared for, not only for their own sakes, but in order that they might carry on their work with efficiency. The rooms which had now been added to the building, consisting of 24 bedrooms, sitting-room, bath rooms, etc., would, he hoped, be in every way suited to the requirements of the nursing staff. They had been erected at a cost of something like £11,000 or £12,000. New isolation wards and servants' quarters had also been arranged; a new passenger lift had been constructed, and new heating apparatus put in for warming the corridors. The telephone had been installed on each floor; the kitchen had been refloored; an escape staircase had been constructed for the new floor; Teale grates had been put into the wards, and the whole interior had been repainted and the outside brickwork repointed. A room had been set aside for the Rœntgen-ray apparatus, and a radiographer appointed. Thanks to the donation already referred to, the committee had been able to close the year with a deficit of less than £400, after paying architect's and other charges, a very satisfactory state of affairs. He, however, wished to urge upon the friends of the hospital that they must not relax their efforts. It could not be expected that every succeeding year would be as prosperous as that just closed had been. In visiting a children's hospital it was impossible not to be touched by the sight of so much suffering, but there was also a great deal that was encouraging in such a visit. He asked all who were present to do their utmost to make the work of this hospital better known; from its position it ran a risk of being overlooked by the public.

Mrs. de Rothschild having declared the ward open under the new name, Mr. Nestor Tirard proposed that a very hearty vote of thanks be given to Mrs. de Rothschild, and to the lady visitors generally. The work of improving the hospital, although very satisfactory so far, was not yet complete, an urgent and pressing need being a new out-patient department. The present accommodation in that respect was quite inadequate to meet the needs of the institution.

Mr. Leopold de Rothschild in reply paid a high tribute of praise to the authorities of the institution, taking occasion to eulogise the medical and nursing staffs.

The proceedings then terminated, and the visitors inspected the wards and the new floor.

### CENTRAL LONDON OPHTHALMIC.

Mr. Charles H. R. Wollaston, the chairman of the committee, presided on the 25th ult. at the sixtieth annual meeting of the Central London Ophthalmic Hospital, Gray's Inn Road. The attendance was as usual very limited.

In moving the adoption of the report of the committee of management and balance-sheet for 1903, the chairman reminded the meeting that the hospital was situated in a densely-populated district, near three great railway stations, which brought to their doors people from the northern suburbs and from distant parts of the country, while many people from the south also came to them, passing the doors of other hospitals nearer to their homes, and so showing their appreciation of the treatment received at the Central London. They had had 12,964 cases as compared with 12,270 in 1902, while their in-patients had increased from 357 to 365. Accidents and urgent cases numbered 250, and just on 1,000 operations were performed during the year. There had been an increase in their expenditure, due partly to their having treated more patients and to the rise in the price of drugs, but also to the repairs they were obliged to make to the old building. He would press it on all their friends the desirability of rebuilding as soon as possible, but they were resolved not to commence this until they had the bulk of the funds in hand, and this still seemed a long way off, though there had been a substantial increase during the year, and the Building Fund now stood at upwards of £5,900. They were all sorry that Mr. Lister was leaving them, and wished to tender their thanks for all his interest in the hospital. He had had a bath-room fitted up for the nursing staff at his own cost, and had also had the operating theatre repaired and redecorated during the year. The committee were also sorry that Mrs.

Hugall, the matron, was going to retire on account of advancing years. They were much indebted to her, not only for her kindness to the patients, but also for the great care and economy she had always exercised with regard to all the domestic details of the institution. Concluding, he hoped their friends would spare no effort to obtain support for the existing hospital and funds for building the new one.

Colonel Tatham seconded the resolution, which was adopted.

An Honorary Officer on Amalgamation.

Votes of thanks to the honorary officers were passed, and, in replying for the staff, Mr. Brittin Archer referred to the negotiations that had taken place for a scheme of amalgamation with the Royal Free Hospital. These fell through because it was found that the land to be acquired was not in the market as it was thought to be. But there were many good reasons against the absorption of their hospital. The poor recognised that at the special out-patient department of a general hospital cases were handled by ante-graduate students, while in a special hospital they were treated by post-graduate students who had passed their curriculum of five years. That was their reason for crowding their out-patient department and not flocking to the special department of a general hospital. There was great local need for such an institution as theirs, and any attempt to remove it would be very unfair. Their 30,000 attendances would soon drop to 15,000, and before many years to 5,000. They had gone carefully into the question of saving in expenses, but it was found that the only salary saved would be that of the matron—£50 a year. Referring to the recommendation of the Distributing Committee—not the Visiting Committee—of King Edward's Fund that they should amalgamate, it had always seemed to him a pity that it had no advisory aid from gentlemen with special experience of special hospitals. Of course, the general hospitals looked askance at them, because they took a class of case they would like to see at their own doors, but they ought to remember that 50 years ago, when Moorfields, the Westminster Ophthalmic, and their own hospital were in full working order, there were no eye departments to the general hospitals. They must remember that no reduction in the number of hospitals would reduce the amount of work; it would only centralise it, and the more they centralised it the more the work would suffer.

A vote of thanks to the chairman concluded the meeting.

### ROYAL FREE HOSPITAL.

The seventy-sixth annual Court of this hospital was held on Wednesday, 2nd inst. The chair was taken by H. R. H. Princess Christian of Schleswig-Holstein, President, who was supported by Sir Edward Durning-Lawrence, M.P., Mr. Charles Burt, the Mayor of St. Pancras, Mr. F. Layland Barratt, Captain H. M. Jessel, M.P., Lieut.-General Pritchard, C.B., Major Ricardo, Mr. H. Silver, and others. There was a large attendance.

The report and accounts for 1903 were presented by Mr. Burt, treasurer and chairman of the weekly board. After remarking that it was a matter for congratulation that the Royal Free Hospital was neither to be pulled down nor removed, Mr. Burt said the year 1903 had been marked by steady and successful work in every department of the hospital. The total number of persons under treatment in all departments was 42,548, of whom 2,640 were in-patients and 39,908 out-patients, including casualty and maternity cases. During the past three years a census had been taken of all patients in the wards on the second Sunday in January, and it would interest subscribers to know that the number resident in the immediate neighbourhood was 101 in 1903, being a slight increase on the figures for the two previous years. Of the remainder, 32 were from other London districts and 16 from the country, attracted, no doubt, by the reputation of the hospital for medical skill and good nursing. For several years past the committee had received urgent representations of the need for the establishment of an external maternity department in connection with the hospital, both for the sake of the poor women of the neighbourhood and in order that the women medical students might acquire this important part of their instruction without having to attend at other institutions. The department was accordingly opened on July 1, 1903. During the first six months 210 women applied for treatment and 132 cases were attended, all making a good recovery. Two qualified medical women were now employed as obstetric assistants, together with two senior students, for whom temporary accommodation had been found near the hospital. It was estimated that this new development would involve an increased annual expenditure of not less than £300; but he hoped that the great value of the work, both to the poor and to the medical school, would induce new subscribers to come forward and assist the committee to meet the increased cost. With regard to finance, the sum of £17,275 had been received, apart from the Building Improvement Fund. The committee were especially grateful to King Edward's Hospital Fund, which, in addition to the usual annual contribution of £750, made further special grants of £500 for general purposes and £1,500 towards building improvements. These grants were not made without very careful inspection by the visitors on behalf of the Fund, and it might therefore be surmised that they were perfectly satisfied with their investigations. Over £9,000 was received in the form of legacies, constituting more than half the year's income, but, while deeply grateful for this support, the Committee were fully alive to the fact that this source of income was a very uncertain one. He was extremely glad to be in a position to announce that, since the Committee-meeting that afternoon, a cheque for £1,000 had been received from Mr. Baer, one of the trustees of the Annie Zunz Bequest, being a further instalment of the handsome contribution promised (£1,000 for ten years). The total expenditure during 1903 was £14,604, an increase of £1,100 on that of the previous year. As economical managers, the Committee regretted the increase, which was, however, unavoidable, being due to the higher cost of living, the opening of several new departments of work, necessitating increase in staff, as well as to various minor structural improvements effected. One instance of the general rise in expenditure might be cited—viz. nurses' salaries, which had increased during the past ten years from £784 to £1,140 per annum. The recently instituted Roentgen ray and Electrical departments, the largely increased work in the laboratories and museum etc., had caused an additional expenditure of some £600 yearly, without any appreciable increase in the number of patients treated. The Committee had given much careful thought to the question of improvements, including the erection of new operating theatres, but for various reasons, one of which was a dislike to incurring debt, they had had to be postponed. As things were, he was obliged to make an appeal for funds to liquidate the debt of £2,000 on the previous quarter's bills. He was very pleased to announce that, thanks to the efforts of her Royal Highness the President, H.R.H. Princess Louise, as well as all who worked hard to make the bazaar in June a success, the sum of £3,166 was realised, notwithstanding the very unfavourable weather. At the express desire of the President, the whole of that amount was to be devoted to the improvement of the nursing quarters, some of the double-bedded rooms being converted into single ones, and a roof promenade and an electric lift being added, the last-named benefiting the hospital generally. This was a matter on which the President had set her heart; and those who knew the value of the nurses' work would rejoice with her that this useful and beneficent object was rendered possible. The nurses worked very hard, and in his opinion should receive the utmost consideration; and the alterations referred to would add greatly to their comfort. Other necessary improvements, to which the Committee were pledged, were in connection with the sanitary arrangements, towards which King Edward's Fund had contributed £1,500. It was not possible for any hospital in the twentieth century to stand still, and the Committee were anxious to do their best for the noble institution the welfare of which they had so much at heart.

Mr. Silver having seconded the motion, the report was unanimously adopted.

On the motion of Mr. Layland Barratt, the names of H.S.H. the Duke of Teck and the Right Hon. Earl Spencer were added to the list of Vice-Presidents of the hospital.

Captain Jessel proposed, and Mrs. Thorne seconded, a vote of thanks to H.R.H. Princess Louise for her devotion to the interests of the Ladies' Association, to which the President replied that her daughter's services to the hospital were rendered with the greatest possible interest and pleasure.

Mr Davies, in proposing a vote of thanks to the medical staff, referred to the fact that, for the first time in its history, the hospital had upon its staff two women doctors.

Mrs. Scharlieb, M.D., in replying, said the utmost harmony prevailed among the staff. The object of each was the benefit of the sick poor, and of those who would in future come under the ministrations of the young women who came there for their training. She looked upon the work of training doctors as in a real sense an Imperial duty, since a large number of the students who passed through the hospital were destined for foreign stations. The hospital intended to be second to none in efficiency as a training school.

Votes of thanks to the matron and nurses, the secretary and other officers, and to H.R.H. Princess Christian for her great service to the hospital in organising and personally assisting from day to day in the bazaar of June last, and for presiding over the Court, closed the meeting.

### ROYAL WESTMINSTER OPHTHALMIC HOSPITAL.

THE annual meeting of this hospital was held on Friday 4th inst., Sir Charles Turner presiding. The Treasurer, in presenting the statement of accounts, said the income of the General Fund in 1903 was £2,495, against £2,797 in the previous year, exclusive of legacies and the grant of £400 from King Edward's Hospital Fund. The hospital had been fortunate in receiving legacies to the amount of £1,323, and this sum, with the grant just referred to, had been carried to the General Purposes Account to pay off the loan necessarily incurred in the alterations effected in 1902. The original cost amounted to £9,189, and in satisfaction of this £5,200 was raised on loan, and the balance provided by special donations and the sale of stock. The debt to the bankers had been reduced to £2,000, to which result grants from King Edward's Hospital Fund, amounting in the aggregate to £900, had materially contributed. It was, however, highly desirable that the debt should be entirely wiped off, and he would urge this upon the friends of the hospital. The ordinary expenditure was £2,526, as against £2,597, and called for no special remark. He thought it very satisfactory that for this sum they had been able to relieve 743 in-patients and 11,395 out-patients, as had been done during the year under review. That this had been possible was largely due to the increased facilities for treatment afforded by the alterations already alluded to.

The Chairman said that during 1903 progress had been made in the amount of work accomplished in all departments of the institution. There was a record number of out-patients, showing an increase of nearly 2,000 on the figures for the previous year. The numbers naturally fell off during the alterations, but they had not only recovered their former level, but had somewhat exceeded it. The value of the hospital was self-evident, and he did not think any in London could show better or more adequate arrangements for the treatment of the special diseases for which it had been founded. He regretted to have to state that in April last Miss Milburn resigned her position as matron, after 6½ years' service; her place had been taken by Miss H. Shorto, formerly assistant matron at the Royal Portsmouth Hospital, who, the Committee had every reason to believe, would maintain the administration of the nursing department at the level of excellence to which it had been brought by her predecessor. He congratulated the Governors on the fact that, notwithstanding the general financial depression from which most charitable institutions had recently been suffering, and the necessity to which the Committee had been put of appealing for help towards the liquidation of the debt on the building fund, the year's income had been maintained very nearly at the figure of the preceding year. He hoped the special effort required to completely wipe out the debt would be made, and that each subscriber would undertake to recruit for fresh support, in order that the hospital might continue to meet the needs of the public, and to fulfil the intentions of the founders. He begged to move the adoption of the annual report.

The Hon. Arthur Brodrick seconded the motion, which was carried.

In proposing a vote of thanks to the various officers connected with the hospital, Mr. Brodrick said a great deal of extra work was entailed upon them by the alterations in the building, and he thought the way in which they had acquitted themselves was worthy of the warm appreciation of the Governors.

A vote of thanks to the Chairman concluded the meeting.

### THE NEW HOSPITAL FOR WOMEN.

THE thirty-second annual meeting was held on Tuesday, 8th inst., her Grace Adeline, Duchess of Bedford, in the chair. There were also present Sir William Broadbent, Dr. Cullingworth, Mrs. Scharlieb, M.D., M.S.; Mrs. Boyd, M.D.; the Rev. H. L. Paget, and others. There was a large attendance.

The Chairman, in proposing the adoption of the report and accounts for 1903, expressed her deep sympathy with the aims and scope of the hospital, which provided poor women with medical and surgical treatment by physicians and surgeons of their own sex—a *sine quâ non* with many women in all ranks of society. It was deplorable that the hospital contained only 52 beds, and that the pressure upon the out-patients' department was daily increasing without prospect of immediate extension to meet the growing demand. There was also grave need for a small special ward for the performing of minor operations, for there were many cases in which prompt treatment resulted in restoration to health, and this could not so conveniently be given in the patient's home. She commended the establishment of such a ward as a form of charity calculated to benefit a large number of poor women. During the year a generous donation of £1,000

had been received from the Misses Garrett for the endowment of a bed which, at their desire, had been named "Patience." They had expressed the wish that those who occupied it might, if necessary, have the benefit of treatment for a longer period than was usually available for the ordinary hospital patient. She urged upon those present the necessity of making the hospital and its work more widely known, and suggested that a concentrated effort should be made to endow another bed, which, as the outcome of such an effort, might fitly bear the name "Purpose."

Sir William Broadbent seconded the motion. The hospital, he said, had so well justified its existence that he felt it to be a great honour to be a member of the consulting staff. He had joined it originally with a view to helping a young and struggling institution; now, however, the hospital had become independent to a large extent, and it was time that the staff were enabled to take the place to which they were entitled in the medical and scientific world. In order to do this it was an absolute necessity that the pathological department should be completely equipped, for the special facilities for scientific investigation possessed by the hospital indicated development along the lines of research. Problems in chemistry, microscopic work, and bacteriology, requiring infinite patience, manipulative skill, and minute observation and intelligence, were eminently suited to the capacities of medical women, and on these grounds he urged that the hospital, already fully equipped for clinical work, should be made complete in the pathological department.

Dr. Cullingworth dwelt on the importance of the hospital as a factor in medical education, and said he hoped to see the day when the number of beds would be sufficient for the entire course to be gone through, without the necessity, on the part of students and nurses, of completing their training elsewhere.

Mr. Henderson, in proposing a vote of thanks to the auditor, Mr. E. Trenow, drew attention to the financial needs of the hospital, which owed £354 on bills unpaid at the close of the year. Apart from legacies, the receipts were £5,758, and there was an adverse balance of £35 after paying the sum of £274 owing from 1902, and the paving of the courtyard, which cost £200. The committee were anxious not only to maintain the hospital at its present level, but to extend its scope, and for this additions to the ranks of the subscribers was an urgent necessity. Mr. Trenow deserved their warm thanks for his gratuitous services in examining the accounts.

The retirement of Mrs. Garrett Anderson from active participation in the affairs of the hospital was alluded to in terms of regret by all the speakers; Dr. Cullingworth, in proposing the re-election of the committee, remarking that Mrs. Anderson was not only the foundress of the New Hospital, but the pioneer in England of the movement for securing the admission of women to the medical profession. Mrs. Anderson retains her place on the consulting staff.

A vote of thanks to the Duchess of Bedford, proposed by Mrs. Scharlieb, and seconded by the Rev. H. L. Paget, closed the proceedings.

### SAMARITAN FREE HOSPITAL.

The fifty-seventh annual meeting was held on Tuesday, 8th inst., the Rev. Dr. Oxford presiding. The chairman presented the report, stating that the year 1903 opened with a deficit of £1,465, composed of a banker's loan of £1,000, an overdraft making up the total. Annual subscriptions showed a decrease of £20 10s., but donations were £918 higher than in the previous year, and legacies showed an increase of £708. Through the kind agency of Dr. Patmore Sheehy and Dr. Percy Boulton, a sum of £1,000 was assigned to the hospital by the executors of the late Mr. Coventry Patmore, and this had been placed to the credit of the Endowment Fund. Additions to this fund were urgently needed. The three distributive funds had made grants on the same high basis of award as heretofore, showing their appreciation of the way the work was carried on. One hundred and eighty-four of the in-patients admitted during the year came from the provinces; the total number was 528, as against 495 in 1902. The number of out-patients had risen from 6,737 in 1902 to 6,796 in 1903, and an appeal was being issued to the public to enable the committee to build a new out-door department. It was hoped that building operations might begin at midsummer of the current year, when the adjoining site would come into possession of the hospital. Included in the building scheme were a new operating theatre, structural alterations so as to make room for a larger number of patients, and additional accommodation for the nurses, the sum required being £20,000.

Mr. Richard Wheen having seconded the motion, the report was adopted.

On the motion of Dr. Owen, seconded by Colonel Watson, it was resolved to omit the words "and children" from the official title of the hospital, cases of the admission of children being extremely rare.

